# Strategies for creating undergraduate research and outreach experiences

**DOI:** 10.1186/s12919-025-00334-4

**Published:** 2025-06-24

**Authors:** Fadie T. Coleman, Danielle E. Graham

**Affiliations:** 1 Department of Oral Medicine, Infection, and Immunity, Harvard School of Dental Medicine, Boston, MA USA; 2 Department of Biological and Forensic Sciences, Fayetteville State University, Fayetteville, NC USA

**Keywords:** Research experiences, Teaching, Outreach, Undergraduate science curriculum

## Abstract

Engaging students in research and outreach offers a range of benefits, including acquiring scientific knowledge and critical skills, fostering science identity development and increasing persistence in science, and providing broad exposure to STEM careers. Although undergraduate research experiences have been shown to be impactful for developing scientists, exploring ways in which all students can experience these opportunities is an important focus that merits continued discussion. This paper will discuss ways in which well-known strategies for incorporating research into science curricula can be further adapted to have a broader impact on undergraduate education and science outreach programming. In addition, key insights inspired by an “Adapting Your Research for Teaching and Outreach” workshop will be considered. Lastly, this paper will discuss ways in which inclusive strategies can facilitate practical application across diverse educational settings.

## Background

When exploring mechanisms for expanding research exposure in science training, it is important to consider practices that are already in place to build on existing knowledge and identify areas for improvement. Engaging undergraduates in the “high impact practice” [1, 2] of research is considered critical to the comprehensive training of scientifically literate students and future generations of scientists [3–6]. Strategies towards engaging undergraduate students in science practice [7–10] include: Capstone [11–15] projects, independent studies [16], summer research experiences (SREs) [17], Undergraduate Research Experiences (UREs) [3, 5–7], Course-based Undergraduate Research Experiences (CUREs) [18], and variations on these pedagogical frameworks [4, 19–23]. A particular highlight on CUREs will allow for a focused discussion on approaches for adapting research for undergraduate teaching and outreach. The previously mentioned strategies offer effective, feasible, and thoughtful ways of integrating hands-on research practice into the undergraduate curriculum to enhance scalability. However, there is still a need and interest to broaden such access to underrepresented groups, such as historically excluded persons (PEERs, persons excluded because of their ethnicity or race [24]) in science. Here, we discuss how undergraduate research experiences that have been adapted for course teaching (such as CUREs [18]) can extend beyond the classroom through outreach efforts. Through these outreach opportunities, the sharing of knowledge and resources with a broader community are made possible. This particular focus on CUREs is geared toward encouraging a broad range of educators to consider incorporating research into their curriculum as a pedagogical tool to further enhance critical science workforce skills, scientific literacy, and science career exploration in both the classroom and non-traditional learning environments. This article is inspired by information shared during the 2019 and 2020 NIH-ASCB Accomplishing Career Transition (ACT) webinars, “Adapting Your Research for Teaching and Outreach,” led by Dr. Erin L. Dolan (former editor-in-chief of CBE—Life Sciences Education). The ACT program is funded by the National Institute of General Medical Sciences Innovative Programs to Enhance Research Training (IPERT) initiative and led by the American Society for Cell Biology (ASCB), which supports professional development and training for postdocs and assistant professors in the biological and biomedical sciences.

As an extension of Dr. Dolan’s “Adapting Your Research for Teaching/Outreach in Biology” webinar, this paper highlights the importance of integrating research into undergraduate biology education and brings awareness to established strategies for adapting research to teaching and some practical methods for impactful outreach. While the webinar introduced foundational ideas, this article expands on those concepts by discussing the benefits of making research experiences accessible to all students. This article also offers considerations around issues of accessibility, equity, feasibility, and potential challenges to support thoughtful planning for effective programming. The focus on outreach is to discuss ways in which access, awareness, education, and sense of belonging can be further enhanced for students in STEM. Lastly, this paper also provides practical suggestions to enhance sustainability for continued inclusive practice and success. Although this article mainly focuses on undergraduates, both STEM and non-STEM majors, the central theme is fostering a culture where research becomes a cornerstone of education and provides a framework for educators seeking to transform their teaching and outreach using approaches that enhance access, equity, and foster inclusive research experiences for all levels.

### Integrating research into undergraduate science curricula

A review of the literature for effective approaches to engage students in scientific inquiry reveals the impactful educational practice of directly connecting research and teaching in the academic curricula [1, 25–30]. Accepted strategies for incorporating research into the undergraduate curriculum encompass a spectrum of approaches [31] (Table 1). One method involves infusing research components (e.g. problem solving, communication, and information synthesis) [32] into existing courses and introducing students to research methodologies within familiar contexts. Capstone projects culminate academic journeys and challenge students to undertake extensive research projects that showcase their acquired knowledge and skills [11–15]. Independent study opportunities allow motivated students to explore specific research topics under the guidance of research mentors [16] at their undergraduate institutions or local research centers. Summer research opportunities [17] offer intensive research experiences during intersession breaks. Service-learning research projects address real-world issues through collaborative partnerships with community organizations [33]. Such research experiences can be impactful in training students interested in pursuing a science career because they help impart a sense of accomplishment [34, 35]. Incorporating research into undergraduate science curricula offers many benefits that greatly enhance the overall educational experience [36–38]. Connecting research and teaching cultivates an intellectually stimulating learning environment [18], prepares students for advanced studies, fosters student readiness for the collaborative demands of academia and the scientific workforce industry, and provides real-world training for future careers in research-intensive fields [39].

**Table 1:** Summary of various approaches for integrating research into undergraduate science curricula (including things to consider for application)

APPROACH	DESCRIPTION	THINGS TO CONSIDER
**Capstone Project**	A summative exercise designed to capture the culminating experience in a student’s academic training (typically includes the evaluation of both didactic and skill-based learning) [11–15]	▪ Fostering collaboration across disciplines can enhance exposure to diverse perspectives and comprehensive problem-solving skills ▪ Encourage project designs that involve the application of both theoretical and real-world scenarios ▪ Orchestrate structured mentor–mentee relationships (between faculty/experts and students) that include opportunities for a productive exchange of feedback
**Independent Study Opportunities**	Individualized learning opportunities that offer students the option of conducting self-directed projects on a specific topic of interest under the supervision of a faculty mentor (higher level research opportunity with a great deal of independence) [16]	▪ Frontloading guidance for students can support the development of a clear research plan and timeline to address the chosen research question/problem ▪ Encourage students to select research topics that align with their interests and mentor expertise (to enhance engagement in the chosen project) ▪ Provide opportunities for students to effectively communicate their research progress, findings, and challenges (e.g. written reports, presentations, and school sponsored Research Days/Symposiums
**Summer Research Opportunities**	Intensive summer opportunities for undergraduate students (typically lasting 8–10 weeks) to engage in hands-on scientific inquiry under the mentorship of faculty members, either at their home institution or at another institution [17]	▪ Designing research opportunities that provide all students (regardless of background) with practical, hands-on experience with conducting experiments, collecting data, and analyzing findings allows for greater outreach impact ▪ Encourage specialized training for mentors (focused on effectively work with diverse student populations) to foster an inclusive and supportive research environment for students ▪ Offer opportunities for students to develop professional networks in an inclusive setting
**Service-Learning Research Project**	Projects that intentionally incorporate community service within academic learning objectives, which allows students to apply course content to real-world issues and consideration of community needs [33]	▪ Developing collaborative relationships with community organizations can help to co-create meaningful service projects that address authentic community needs and maximize the impact of student learning experiences ▪ Encourage the use of diverse teaching methods (including hands-on group work, one-on-one interactions, and open group discussions) to engage all learners and learning styles ▪ Implement a formalized procedure with defined goals to guide the service-learning projects to enhance alignment with educational and community objectives (also supports thoughtful reflection around service experiences)
**Course -Based Undergraduate Research Experience (CURE)**	Research experiences are incorporated directly into undergraduate courses, allowing students to engage in authentic scientific inquiry as part of their curriculum (students apply course content to real-world problems, and contribute to advancing scientific knowledge) [36–38]	▪ Use of a backward-design approach can help to integrate research goals within course learning outcomes (to highlight relevance and course content alignment) ▪ Encourage the use of active learning strategies that promote student engagement and collaboration (such as inquiry-based activities, group projects, and hands-on laboratory work) to enhance problem-solving skills ▪ Provide comprehensive mentorship for students throughout the CURE, offering guidance, resources, and opportunities for reflection to enhance research skills and support academic success and career development [27, 28, 39]

Common among the different approaches for integrating research into undergraduate learning experiences is the emphasis on feasibility. The successful execution of undergraduate research experiences hinges on access to: 1-effective mentorship from faculty mentors with expertise in research methodologies and practices, 2-adequate resources, 3-funding, 4-well-equipped laboratory facilities, and 5-research tools. Additional considerations include an appreciation for the time commitment involved in research training. Balancing research commitments alongside regular coursework necessitates meticulous student and faculty time management. An interdisciplinary approach that involves collaboration between different academic departments can amplify available resources and expertise, enhancing the feasibility of integration. Adaptation to the institution’s curriculum structure is also crucial, allowing research integration to vary from minor components within existing courses to comprehensive capstone experiences [27, 28, 39].

### Making undergraduate research experiences accessible to all

Making research accessible to all requires a focus on creating inclusive environments [33], such that a broad diversity of people feel welcome and have access to learning opportunities in research to learn, share perspectives, and thrive [40]. The importance of students seeing themselves as scientists, who can contribute to the advancement of science, is critical to the leading global status of our nation’s economy [25, 41–44]. Therefore, undergraduate programs and initiatives that support student success in STEM should include an intentional and targeted strategy for enhancing learning in STEM fields by fostering positive perspectives around career attainment and potential contributions to the field [45–47]. These experiences are especially crucial for PEERs [24] and students belonging to groups underrepresented in medicine [17]. National reports show that U.S. health professions and scientific research communities do not reflect the nation’s diverse demographics [48]. Therefore, making undergraduate research experiences accessible to all is crucial in promoting inclusivity and diversity in higher education and the overall science community [48–51]. For example, to enhance accessibility, institutions could consider a flexible framework that includes a broad range of research and interdisciplinary projects that can be adapted for teaching (such as community-based research [33]) and also allows students to grapple with real-world issues [52–56].

#### CUREs and their strengths in establishing equity for all students

Undergraduate research experiences embedded within courses are pivotal in establishing student equity [33]. These experiences offer an inclusive platform that levels the playing field, since they are integrated into the curriculum and accessible to every student. By weaving research components into coursework, students can get exposed to research methodologies in a scaffolded manner, which promotes student engagement with scholarly activity while narrowing existing knowledge gaps due to disparities in access or exposure to prior research opportunities [18]. The strengths of CUREs in promoting equity are manifold [18, 57]. They dismantle the barriers that may hinder students from marginalized backgrounds by providing equal access to research opportunities [57]. CUREs also offer a supportive and structured environment, which can be beneficial for students new to research [58]. Through close faculty mentorship and collaboration, students receive guidance that can help bridge knowledge gaps and build confidence [59]. Furthermore, CUREs create an active culture of inclusivity and diversity in academia [57]. They emphasize the value of every student’s contribution, irrespective of their academic trajectory, fostering a sense of belonging. These experiences align with pedagogical approaches that prioritize active learning and student-centered education.

#### Research and teaching’s potential for societal impact

The practice of incorporating research into the science course curriculum is significant. Teaching research in the classroom takes science beyond the didactic, exposing students to critical skills to conduct hands-on scientific investigation. This student engagement with science content can develop an appetite for pursuing research careers [1]. Studies in science education research continue to support the implementation of undergraduate research programs into the science curricula [3]. The development of critical thinking skills and effective problem-solving skills are just some of the benefits that can be gained from introducing hands-on research experiences in the classroom. For some students, this is the only exposure to the entire research process, where they can understand how research informs the field through sharing of results and their interpretations. The compelling need and potential for societal impact underscores the imperative to adapt research for teaching and outreach. This adaptation is particularly crucial due to the growing diversity of classrooms [48, 60], which calls for greater inclusion and consideration of various backgrounds, perspectives, and learning styles. When discussing the impact, it is important to consider whom this adaptation will affect, such as students from different cultural, ethnic, and socio-economic backgrounds and those with varying cognitive abilities [50]. In essence, the goal is to ensure that research-based teaching and outreach initiatives resonate with and engage all members of society.

#### Growing diversity of classrooms calls for greater inclusion

Reaching a broader audience necessitates innovative approaches. Instructors and educators can employ culturally relevant teaching methods [61], interactive activities, and digital platforms to make research concepts more accessible and relatable. This approach should involve using real-world examples that resonate with diverse groups of students [62], breaking down complex concepts into digestible components, and creating a more inclusive learning environment [62] where every voice is valued. Adapting research for teaching and outreach in this manner ensures that knowledge dissemination transcends traditional barriers and actively involves a broader cross-section of the population [57]. Additionally, maintaining global leadership status in science requires the proper training of developing scientists [60, 63].

When effectively integrated into teaching and outreach, research cultivates a culture of persistence and dedication in the scientific community. Research can be incorporated into outreach through participatory research, where community members pose questions, contribute to data collection, and collaborate on solutions, fostering mutual learning and trust. Partnerships between scientists and educators can also address disconnects in K-20 + science education, enabling both groups to refine their practices and perspectives [64]. Outreach can also involve demonstrations of research activities to showcase findings and their real-world applications, bridging the gap between science and community understanding. Students exposed to research early in their training have the potential to develop a strong foundation and passion for science [65, 66].

Archer and Bourdieu’s theory of science capital suggests that students with higher levels of human, cultural, and social capital are more likely to have access to research opportunities. When these students engage in research, they further develop these forms of capital, creating a positive feedback loop that enhances their learning, skills, and confidence in science. Consequently, students who enter college with ample science capital are more likely to excel in research experiences, fostering a strong foundation and passion for science early in their training [67–72]. Therefore, equitable science training creates opportunities for individuals from all backgrounds to excel within the scientific workforce. Scientific knowledge is most successfully realized and communicated by diverse scientists and teams. Inclusivity enhances productivity and fosters the creation of innovative solutions to global challenges, as diverse perspectives bring a richness of ideas and approaches.

#### Considering high impact educational practices with an equity lens

Historically in higher education, research and teaching have been recognized as separate entities. Traditional models of the relationship between research and teaching indicate that research generates knowledge, and teaching transmits such knowledge to students [73, 74]. Adapting research for teaching and outreach is a multifaceted endeavor that can be guided by high impact educational practices and an equity lens [33, 75–78]. In this approach, active learning methods accommodate various learning styles, while integrating diverse perspectives and voices into research content and teaching materials, which fosters inclusivity [79]. Moreover, understanding the nuances of different educational environments, such as Primarily Undergraduate Institutions (PUIs), Minority-Serving Institutions (MSIs), and Research 1 (R1) Institutions, is pivotal. Tailoring strategies to each context ensures that research-driven education resonates effectively, whether by prioritizing hands-on experiences at PUIs or aligning research initiatives with the unique needs of communities served by MSIs [57].

A list of potential challenges with incorporating research in the classroom are included in Table 2. Awareness of such challenges and relevant considerations can inform critical conversations and generate creative approaches for successful implementation. For example, students have noted concerns with integrating research and teaching, including limited availability of professors, exclusion from the research process, or not seeing themselves as stakeholders in the research [80, 81]. Course instructors can consider making course content related to research topics that are interesting and relevant to students to further support student involvement and success [81]. Identifying resources and securing institutional support play pivotal roles in successful adaptation [82, 83]. Seeking funding opportunities for curriculum development, community engagement, and student research experiences helps fuel these initiatives. Collaborating with academic leaders to raise awareness about the significance of research-based teaching and outreach efforts can garner the necessary support. Additionally, establishing faculty development programs offers educators the training to seamlessly integrate research into their teaching practices, enhancing the overall learning experience.

**Table 2:** Considerations for addressing some common challenges with integrating research into teaching

**Challenges**	**Considerations**
**Limited Resources, Funding, and Infrastructure for Research** [81, 82]	▪ Seek external grants and internal funding opportunities to support teaching-research objectives ▪ Establish cross-institutional research-teaching partnerships ▪ Leverage technology to enable virtual laboratories, remote access to equipment, and collaborative research platforms to overcome physical constraints
**Competing Priorities (e.g. Faculty Workload)** [79, 80, 84, 85]	▪ Incentivize integration of research into teaching practices to apply to personnel professional advancement (tenure, promotion, reappointment, or merit-based raises) ▪ Establish STEM course research-based experiences ▪ Implement outreach programs (i.e. service-learning projects) into academic courses
**Limited Faculty Volunteer Pool** [79, 80]	▪ Foster interdisciplinary collaborations ▪ Provide stipends for faculty engaged in research-based teaching ▪ Provide professional development to enhance faculty skills in pedagogical techniques that incorporate research into teaching
**Diverse Levels of Academic Preparedness** [27, 28, 39]	▪ Review and consider revising curriculum to better align with research-based teaching practices ▪ Provide co-curricular activities that build on research experiences and provide opportunities for applied practice
**Enhancing Accessibility and Inclusivity** [48–51]	▪ Implement universal design for learning (UDL) principles to create teaching practices and materials that are accessible to all students ▪ Collaborate with various campus divisions such as learning specialist or disability support services, instructional designers, and experts to provide resources, training, and guidance on how to make research-integrated teaching practices more accessible and inclusive

A collaborative approach further amplifies the impact of research-driven teaching and outreach endeavors. Collaborating with other institutions enables sharing of best practices, resources, and experiences, fostering a collective impact [86]. Forming consortia or partnerships facilitates the pooling of expertise and creates a synergy that benefits all involved. The joint development of curriculum, collaborative workshops, and interdisciplinary initiatives expands students’ exposure to diverse perspectives and fosters a sense of unity in pursuing educational excellence. By weaving these strategies together, educators can adapt research for teaching and outreach in a manner that leads to transformative impact. This approach promotes equity and can be applied to various educational contexts. It can help secure vital institutional support and harnesses the power of collaboration to create an inclusive educational experience for all.

#### Extending high impact educational practices to the community

Outreach often refers to organized efforts to engage audiences, such as students, educators, and the public in activities that enhance their understanding of science and research through hands-on experiences, mentorship, and educational programs [64]. In this context, outreach primarily targets undergraduate students to provide exposure to scientific fields and foster interest and skills that may inspire advanced educational or career pathways. Outreach programs can play a pivotal role in shaping the educational journeys of students and trainees, regardless of their background or expertise [86]. They provide transformative opportunities that ignite a passion for science and research and nurture the development of thought leaders who can drive innovation and change. By enhancing science literacy and fostering practical communication skills, outreach initiatives ensure that the next generation of scientists not only advances the frontiers of knowledge but also contributes to a more informed and engaged society. These opportunities can be particularly impactful for students from underrepresented groups, as they offer exposure to unfamiliar fields and provide opportunities that can shape all students early in their training [83]. By engaging students in hands-on experiences, these programs foster critical thinking, problem-solving, and scientific inquiry. Participants learn the technical aspects of research and how to approach challenges creatively and collaboratively. This training equips students with the skills to address complex scientific questions that encourage the development of visionary leaders in their respective fields [83].

To extend the impact of outreach initiatives and promote best practices within the community, it is essential to establish partnerships with stakeholders beyond one’s current position. This involves actively seeking opportunities to collaborate with groups or individuals who have shared interests and objectives towards common goals [84]. Additionally, providing early opportunities [86, 87] for all students and trainees to engage in science-related activities fosters leadership development and community engagement. The community includes K-12 students, educators, and families. These outreach programs emphasize partnerships between universities and the community to provide authentic scientific experiences, foster connections with diverse role models, and align efforts with mutual goals. Effective partnerships involve actively engaging stakeholders by asking what they truly need, clearly communicating what the university can offer, and identifying common ground to ensure programs are accessible, relevant, and inclusive. By building on shared interests and fostering meaningful engagement, such collaborations strengthen community ties and create opportunities for sustained impact [86]. Encouraging participation in science clubs and offering mentorship to students not only enhances their learning experiences but it also cultivates leadership skills and promotes a sense of responsibility to the community. Moreover, adopting strategies to effectively communicate scientific findings and engage with diverse audiences, such as through citizen science projects and assisting students with science fair projects, contributes to building credibility and trust within the scientific community while fostering meaningful connections with the wider society [85, 88].

In an era where scientific information is abundant yet easily misinterpreted, effectively communicating research findings is crucial. Outreach initiatives emphasize the art of conveying complex scientific concepts to diverse audiences, bridging the gap between researchers and the public. By honing their communication skills through interactions with schools, community groups, and the public, students learn to articulate their research in a way that is accessible and engaging to non-experts [89]. Integrating research into undergraduate curricula and highlighting the benefits of hands-on research experiences supports the development of critical skills and workforce competencies.

The concept of adapting research for teaching and outreach invites educators to explore practical strategies for modifying research projects so they can be taught in course settings. The focus on outreach efforts highlights the potential course-based research experiences possess to expand into non-traditional classrooms and diverse learning environments. Initiatives that fall under the umbrella of outreach are poised for adaptation and provide a tailored approach to meet specific needs or address specific gaps in access. Table 3 displays some helpful approaches for designing and developing niched programming, including examples of established research science pedagogy and effective practices, considerations for enhancing student engagement, and thoughts on enhancing diversity and inclusion, and broadening participation.

**Table 3:** Ten helpful approaches for adapting research for teaching and outreach

Helpful Approaches for Adapting Your Research for Teaching and Outreach
1. Incorporate undergraduate research experiences into the academic curriculum (e.g. Capstone projects, independent studies, summer research experiences (SREs), Undergraduate Research Experiences (UREs), and Course-based Undergraduate Research Experiences (CUREs)) [31]
2. Provide opportunities for students to gain effective mentorship, access to resources and well-equipped laboratory facilities to further support successful undergraduate research experiences [27, 28, 39]
3. Consider collaborating across academic departments to amplify resources, funding, expertise, feasibility [81–83]
4. Foster inclusive environments that support all students in envisioning themselves as scientists, especially underrepresented groups [33]
5. Offer flexible frameworks and interdisciplinary projects (such as community-based research) to enhance accessibility [33]
6. Utilize coarse-based research experiences as a platform for broadening participation, providing equal access to research opportunities, and offering structured support and mentorship to students from diverse backgrounds [18, 57]
7. Acknowledge the time commitment involved in research training and support students and faculty in balancing research commitments alongside regular coursework [28]
8. Cultivate student leadership in science through mentorship, outreach, and engagement in community-based research initiatives, fostering a sense of ownership and contribution [60]
9. Enhance science literacy and communication skills through outreach programs, bridging the gap between researchers and the public and fostering a more informed and engaged society [82]
10. Collaborate across institutions and form alliances to share best practices, resources, and experiences, fostering a collective impact approach that benefits all stakeholders and enhances the educational landscape [86]

In summary, adapting research for teaching and outreach presents a transformative approach that enhances science education in a multi-dimensional fashion. This paper emphasizes the benefits of making research experiences accessible to diverse cohorts, adapting research for teaching that can accommodate different skill levels, and creating equitable science training by extending research to diverse outreach efforts (Table 3). The imperativeness of adapting research for teaching and outreach is driven by a dual commitment to establishing equity and enhancing societal impact in STEM training. This commitment is spurred by the burgeoning diversity within classrooms and the compelling necessity to extend research-based education’s accessibility, even within conventional academic structures. Creative approaches, encompassing culturally relevant teaching methods and interactive pedagogies, emerge as pivotal tools for making intricate research concepts comprehensible to diverse learners. Additionally, the paper highlights this approach’s profound role in cultivating the forthcoming generation of scientific leaders, instilling attributes of resilience, dedication, and a firm scientific foundation. The need for equitable science training is imperative, not only for enhancing economic productivity but also for catalyzing solutions to global challenges. This article underscores the need for creativity, collaboration and collective impact approaches from institutional alliances to foster a more comprehensive and impactful educational landscape.

## Data Availability

Not applicable.

## Abbreviations

CURE: Curriculum-based Undergraduate Research Experience
PEERs: Persons Excluded because of their Ethnicity or Race
SRE: Summer Research Experience
STEM: Science, Technology, Engineering, and Mathematics
URE: Undergraduate Research Experience
MSIs: Minority-Serving Institutions
PUIs: Primarily Undergraduate Institutions
R1s: Research 1 Institutions
ACT: Accomplishing Career Transition
ASCB: American Society for Cell Biology
IPERT: Innovative Programs to Enhance Research Training
NIH: National Institutes of Health
